# Population immunity to varicella in Canada: A Canadian Immunization Research Network (CIRN) study

**DOI:** 10.1371/journal.pone.0309154

**Published:** 2024-08-19

**Authors:** James Wright, Natasha Crowcroft, Elizabeth McLachlan, Carol Perez-Iratxeta, Eugene Joh, Selma Osman, Todd Hatchette, Shelley L. Deeks, Sarah E. Wilson, Stephanie L. Hughes, Scott A. Halperin, Sarah A. Buchan, Brian J. Ward, Jonathan Gubbay, Marc Brisson, Bouchra Serhir, Alberto Severini, Shelly Bolotin

**Affiliations:** 1 Public Health Ontario, Toronto, Ontario, Canada; 2 Centre for Vaccine Preventable Diseases, Dalla Lana School of Public Health, University of Toronto, Toronto, Ontario, Canada; 3 Dalla Lana School of Public Health, University of Toronto, Toronto, Ontario, Canada; 4 Department of Laboratory Medicine and Pathobiology, University of Toronto, Toronto, Canada; 5 National Microbiology Laboratory Branch, Public Health Agency of Canada, Winnipeg, Manitoba, Canada; 6 Statistics Canada, Ottawa, Ontario, Canada; 7 Canadian Center for Vaccinology (CCfV), IWK Health Centre, Nova Scotia Health Authority (NSHA), and Dalhousie University, Halifax, Nova Scotia (NS), Canada; 8 Community Health and Epidemiology, Dalhousie University, Nova Scotia, Canada; 9 Nova Scotia Department of Health and Wellness, Halifax, Canada; 10 Departments of Pediatrics and Microbiology & Immunology, Dalhousie University, Halifax, NS, Canada; 11 Department of Microbiology and Immunology, McGill University, Montreal, QC, Canada; 12 Département de Médecine Sociale et Préventive, Université Laval, Québec City, QC, Canada; 13 Laboratoire de Santé Publique du Québec/Institut National de Santé Publique du Québec, Sainte-Anne-de-Bellevue, QC, Canada; 14 Department of Medical Microbiology and Infectious Diseases, University of Manitoba, Winnipeg, MB, Canada; Lerner Research Institute - Cleveland Clinic, UNITED STATES OF AMERICA

## Abstract

**Introduction:**

The incidence of varicella in Canada has decreased by almost 99% since vaccination was introduced. However, variation in the timing and eligibility of vaccination programs across the country has resulted in some cohorts being under-vaccinated and therefore potentially susceptible to infection.

**Methods:**

We used nationally representative specimens from the Biobank of Statistics Canada’s Canadian Health Measures Survey (CHMS) as well as residual specimens from Ontario collected between 2009–2014 to estimate population immunity across age-groups and geography, and identify any groups at increased risk of varicella infection.

**Results:**

The weighted proportion of specimens with antibody levels above the threshold of protection was 93.6% (95% CI: 92.4, 95.0). Protection was lowest among those aged 3–5 years (54.3%; 95% CI: 47.3, 61.4), but increased with age. Individuals born outside Canada had more than twice the odds of varicella susceptibility than those born in Canada (aOR: 2.7; 95% CI: 1.4, 5.0; p = 0.004). There were no differences by sex or geography within Canada, and there were no statistically significant differences when Ontario CHMS sera were compared to Ontario residual sera, apart from in participants aged 12–19 year age-group, for whom the CHMS estimate (91.2%; 95% CI: 86.7, 95.7) was significantly higher (p = 0.03) than that from residual specimens (85.9%, 95% CI: 81.1, 90.8).

**Discussion:**

Varicella immunity in Canada is changing. Children appear to have low population immunity, placing them at greater risk of infection and at increased risk of severe disease as they age. Our results underscore the importance of performing periodic serosurveys to monitor further population immunity changes as the proportion of vaccine-eligible birth-cohorts increases, and to continually assess the risk of outbreaks.

## Introduction

Varicella zoster virus (VZV) is the causative agent of both chickenpox (varicella), most commonly occurring during childhood, and shingles (herpes zoster), most frequently in older individuals. Primary varicella infection is mainly spread by inhalation of aerosols or through contact with skin lesions [1]. VZV is highly transmissible, with varicella household transmission rates among susceptible contacts ranging from 61 to 100% [2]. Infection is often self-limited, however, more serious complications such as pneumonia, encephalitis, and secondary bacterial infections can occur, with a small number of cases resulting in death, more commonly among older or immunocompromised individuals [2].

A live attenuated vaccine against VZV was first licensed in several European countries in the 1980s, and in the United States in 1995 [3, 4]. Use in Canada was approved in 1998 at which time it became available for private purchase [5]. The vaccine was recommended in 1999 by the National Advisory Committee on Immunization (NACI) as a single-dose regimen administered between 12 and 18 months of age [6]. Between 2000 and 2007, varicella vaccination was introduced into the publicly-funded immunization schedule in each of Canada’s ten provinces and three territories at varying times [7]. In 2010, NACI recommended transitioning to a two-dose varicella vaccine regimen due to concerns about breakthrough varicella infection after one vaccine dose caused by both primary vaccine failure and waning immunity [6]. As with the introduction of one-dose regimens, provinces and territories introduced a second varicella dose at different times, starting with Prince Edward Island in 2010, followed by Ontario, New Brunswick, and Saskatchewan in 2011, and other provinces and territories by 2016 (**Table 1**). As part of the National Immunity Strategy objectives for 2016–2021, a target was set to achieve 95% coverage of at least one dose of varicella-containing vaccine in two-years-old by 2025 [8].

**Table 1:** Summary of varicella vaccination rollout across provinces in Canada and what proportion of infants in the analysis cohort would have been eligible for vaccination.

Province	Year of first varicella dose introduction [9]	Year of second varicella dose introduction
**Prince Edward Island**	2000	2010 [10]
**Alberta**	2001	2012 [11]
**Northwest Territories**	2001	^a^
**Nova Scotia**	2002	2012 [12]
**Nunavut**	2002	^a^
**Ontario**	2004	2011 [13]
**New Brunswick**	2004	2011 [14]
**Manitoba**	2004	2014 [15]
**Newfoundland and Labrador**	2005	2013 [16]
**Saskatchewan**	2005	2011 [17]
**British Columbia**	2005	2012 [18]
**Quebec**	2006	2016 [19]
**Yukon**	2007	2012 [20]

^a^: A second varicella dose is included as part of the vaccination schedule in these territories, but the year when this second dose was introduced could not be found

Incidence of varicella in Canada has decreased by almost 99% since vaccination was introduced, from an average annual incidence of 214 cases per 100,000 population in the pre-vaccine era (1993–1997) to an average of 1.6 cases per 100,000 population in 2017 [21]. Over a similar period, pediatric hospitalizations decreased significantly from 398 in 2000 to 42 in 2017 [21]. However, despite clear reductions in varicella incidence, variation in the timing and eligibility of 1- and 2-dose VZV vaccination programs and whether catch-up campaigns were launched, combined with a reduced probability of varicella exposure even for those without vaccine protection, may have led to pockets of varicella susceptibility in Canada.

The primary aims of this study were to assess population immunity to varicella across Canada using sero-epidemiology and identify groups who may be at higher risk of infection. A secondary aim was to compare seroprevalence estimates derived from residual sera in Ontario and sera collected as part of a population-based statistical sample to understand the utility of each specimen source in the Canadian context.

## Methods

We tested serum specimens from the Biobank of Statistics Canada’s Canadian Health Measures Survey (CHMS), a nationally representative survey conducted by Statistics Canada [22]. In this periodic survey, health data were collected from individuals aged 3–79 years residing in all ten Canadian provinces using a combination of household interviews, physical measurements, and blood sample collection. The survey did not collect data on those living in indigenous settlements, those living in the three northern territories, those who serve full-time in the Canadian armed forces, individuals who are institutionalized, or those living in certain remote regions [22, 23]. Samples from both the 2009–2011 survey (Cycle 2) and the 2011–2013 survey (Cycle 3) were combined to ensure an adequate sample size for national and some regional analyses. CHMS data were never accessible to anyone external to Statistics Canada.

In order to compare seroprevalence estimates derived from a statistical sample with those derived from residual sera, we also tested 1,199 sera specimens left over after diagnostic testing from individuals aged 1–39 years from Ontario. We collected the samples between November 2013 and May 2014 from a large private diagnostic laboratory, which conducted a substantial proportion of physician-ordered tests and some hospital testing across the province [24]. These samples were first analysed for the purposes of this study in 2017. Although the residual specimens were anonymized, information was provided on age, sex, and geographic region where the sample was collected (at Forward Sortation Area (FSA) level). Sera were eligible for inclusion if the sample contained a minimum of 2mL, was collected and stored in a serum separator tube, and processed within 48 hours of collection. To minimize selection bias, we aimed to include specimens from across geographic regions in Ontario.

We tested all survey specimens and residual sera using the BioPlex 2200 MMRV IgG assay (Bio-Rad Laboratories, Hercules, California, United States) at the National Microbiology Laboratory in Winnipeg, Manitoba, using an off-label quantitative transformation algorithm, as previously described [13]. We subsequently re-tested those deemed negative (<152 mIU/mL) or equivocal (≥152 and <190 mIU/mL) using a more sensitive glycoprotein-based enzyme-linked immunosorbent assay (gpELISA) (Vacczyme, Binding Site, Birmingham, UK) previously validated by our group [25]. Specimens with gpELISA titres of between 100 and <150 mIU/mL were considered equivocal, while those with titres ≥150 mIU/mL were classified as being above the threshold of protection [25]. For specimens with negative or equivocal results on the BioPlex, the titres were determined by gpELISA which is considered a reference method for VZV immunity testing (**S1 Table**).

The CHMS derives survey weights for estimating seroprevalence and uses bootstrap weights when estimating its variance [26, 27]. In addition, weighting adjustments are implemented to account for non-response bias in the data [22, 23]. In line with Statistics Canada methodology, we assessed the quality of all estimates using the coefficient of variation (CV). CV values of between 16.6 and 33.3 indicated high sampling variability, while those greater than 33.3 were considered unreliable. As per Statistics Canada guidelines, we rounded all weighted estimates to the nearest 100.

To estimate population immunity, we summarized the weighted proportion considered positive, equivocal, or negative by age-group, sex, region, and whether an individual was born in Canada. Due to the CHMS sampling design, whereby there are not enough degrees of freedom available for highly granular geographic analysis [22], provincial analyses were restricted to Ontario and Quebec. As a result, for regional analyses we compared Ontario, Quebec, and “Other” which combined specimens from Newfoundland and Labrador, Nova Scotia, New Brunswick, Manitoba, Alberta, British Columbia, Saskatchewan, and Prince Edward Island. We repeated these summaries with equivocal and negative specimens grouped together, categorized as “non-immune”, and compared with positive sera (“immune”). We assumed a binomial approximation to generate 95% Wald confidence intervals (CIs) for proportions, with Exact methods used if cell counts were below five. We compared proportions between groups using Pearson’s chi-square tests and used a Cochran-Armitage test-for-trend to assess whether a trend was present with respect to age. Additionally, we assessed differences in immunity within age-groups by sex, geography, and whether individuals were born in Canada using Pearson’s chi-square tests with normalized weights, and a Bonferroni correction was used to account for the number of tests conducted. We also calculated weighted geometric mean concentrations (wGMCs) by age-group. We calculated these using gpELISA results when available, otherwise BioPlex results were used.

We used logistic regression to assess unadjusted odds of varicella susceptibility between those born in Canada and those born outside of Canada. We subsequently calculated adjusted odds (aOR) of susceptibility for the same outcome using a logistic regression model that included sex and age-group as *a priori* confounders. Immunity status was defined as immune (above the threshold of protection) or non-immune (either negative or equivocal).

To compare results between CHMS and Ontario residual specimens, we calculated the proportion of Ontario residual specimens above the threshold by age-group and compared them with CHMS samples from Ontario using Pearson’s chi-squared tests.

We conducted statistical analyses in R 4.1.3 and STATA 12.1.

### Ethics

We received ethics approval from the Biobank Advisory Committee of the CHMS, the Public Health Ontario Ethics Review Board, and the University of Manitoba Research Ethics board. Consent from participants was not obtained.

## Results

During cycles 2 and 3, CHMS collected demographic information from 12,180 individuals, with blood samples available for 12,157 of these individuals. For various reasons, including insufficient serum volume and lack of consent for testing, 981 samples could not be tested. We observed the greatest proportion of missing samples among the youngest age-group (3–5 years), females, and those born outside of Canada (all p<0.0001) [28].

Therefore, we tested a total of 11,176 CHMS specimens; a proportional representation of 29,570,500 Canadians. Slightly more than half of the specimens (50.7%) were collected from females, and the majority were collected from individuals born in Canada (80.1%), approximating the composition of Canada’s population (**Table 2**). Just over a third (33.4%) of specimens were collected from Ontario and almost a quarter (22.7%) from Quebec, largely representative of Canada’s geographic population distribution (**Table 2**). Children aged 3–5 years contributed 7.9% of specimens, with the rest fairly evenly distributed between the remaining age bands (see **Table 2** for unweighted and weighted proportions) [28]. A total of 9,296 (83.2%) specimens were deemed positive by BioPlex. The remaining 1,880 specimens deemed negative or equivocal were additionally tested by gpELISA, of which 583 (31.0%) were positive, 1,027 (54.6%) were negative, and 270 (14.4%) were equivocal.

**Table 2:** Characteristics of the study population, from the Canadian Health Measures Survey (CHMS) Cycles 2 and 3, 2009–2013.

	Unweighted (n = 11,176)		Weighted^a^ (n = 29,570,500)	
	n	%	n	%
Sex				
Male	5,513	49.3	14,888,800	50.4
Female	5,663	50.7	14,681,600	49.6
Age-group (years)				
3–5	879	7.9	823,100	2.8
6–11	1,811	16.2	1,830,100	6.2
12–19	1,901	17.0	2,980,300	10.1
20–39	2,269	20.3	8,821,800	29.8
40–59	2,241	20.1	9,698,200	32.8
60–79	2,075	18.6	5,416,900	18.3
Region				
Ontario	3,733	33.4	11,525,000	39.0
Quebec	2,532	22.7	6,902,100	23.3
Other provinces	4,911	44.0	11,143,300	37.7
Born in Canada^b^				
Yes	8,950	80.1	21,856,200	74.0
No	2,224	19.9	7,663,800	26.0

^a^Values rounded to the nearest 100 in line with Statistics Canada policy

^b^Born in Canada information missing for n = 2 individuals

**Fig 1:**
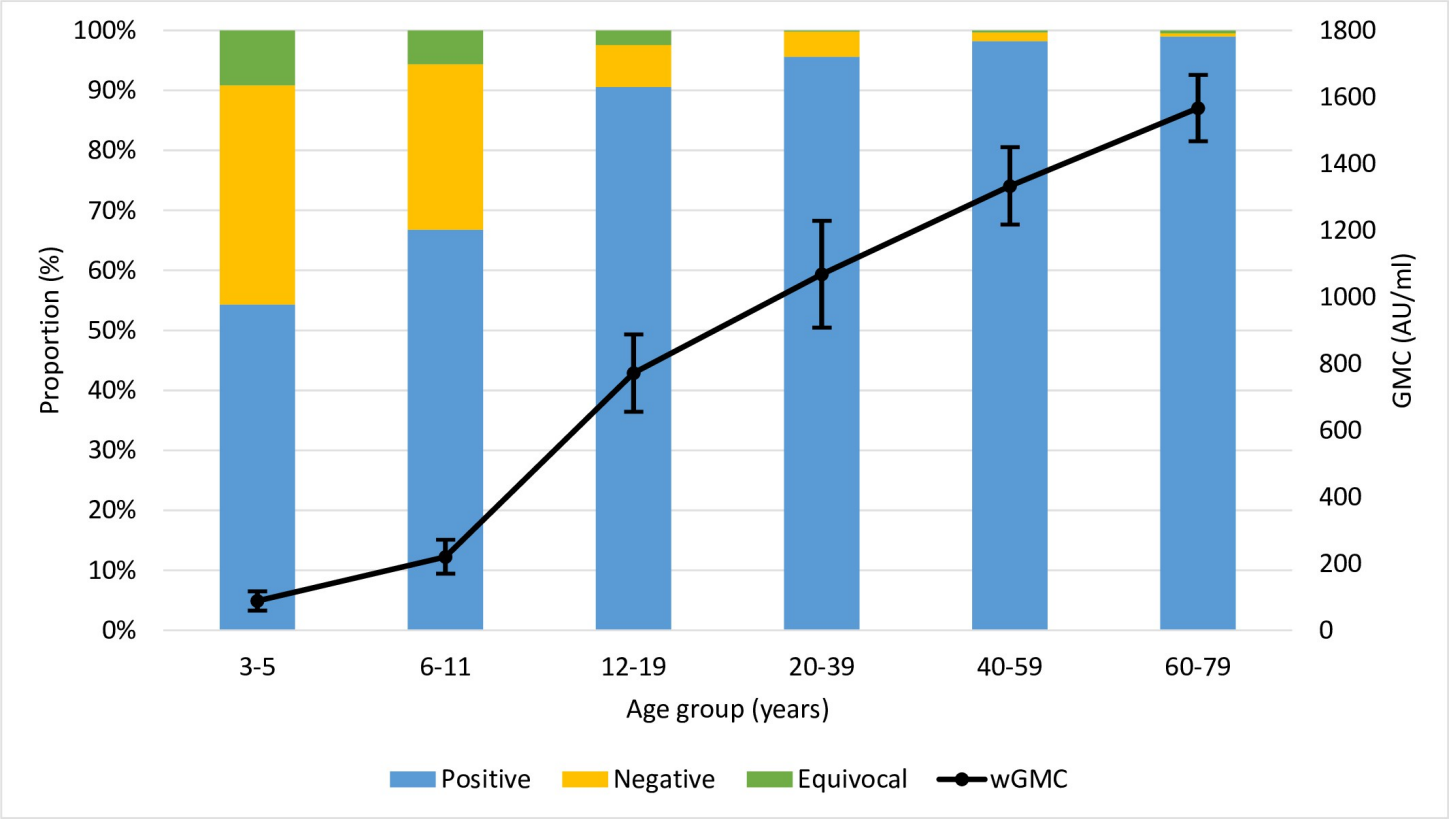
Varicella immunity classification and Geometric Mean Concentration (GMC) by age-group.

Overall, the weighted proportion of specimens with antibody levels above the threshold of protection was 93.6% (95% CI: 92.4, 95.0) while 1.1% (95% CI: 0.8, 1.3) were equivocal, and 5.2% (95% CI: 4.0, 6.5) were negative (**Table 3**). The proportion of specimens with antibodies above the protective threshold was lowest among those aged 3–5 years (54.3%; 95% CI: 47.3, 61.4) and 6–11 years (66.8%; 95% CI: 61.4, 72.2) but increased with age (Cochran-Armitage test-for-trend p<0.001), with all older age-groups showing greater than 90% positivity (**Table 3, Fig 1**). The largest proportion of equivocal results were observed among those aged 3–5 years (9.1%; 95% CI: 6.0, 12.2) and 6–11 years (5.6%; 95% CI: 3.8, 7.4).

**Table 3:** Weighted proportion of samples that were positive, equivocal and negative for varicella antibodies overall and by sex, age group, region and whether born in Canada.

	Positive	Equivocal	Negative	p-value^a^
	% (95% CI)	% (95% CI)	% (95% CI)	
Overall	93.6 (92.4, 95.0)	1.1 (0.8, 1.3)	5.2 (4.0, 6.5)	
Sex				
Male	93.1 (91.3, 94.9)	1.1 (0.7, 1.5)^	5.8 (4.3, 7.3)	0.300
Female	94.3 (92.7, 95.8)	1.1 (0.8, 1.4)	4.6 (3.1, 6.2)	
Age-group (years)				
3–5	54.3 (47.3, 61.4)	9.1 (6.0, 12.2)	36.6 (30.2, 42.9)	<0.0001
6–11	66.8 (61.4, 72.2)	5.6 (3.8, 7.4)	27.6 (22.6, 32.7)	
12–19	90.6 (88.1, 93.1)	2.4 (1.4, 3.4)^	7.0 (4.7, 9.3)	
20–39	95.6 (93.2, 98.0)	0.2 (0.0, 0.4)^#^	4.2 (1.9, 6.5)^	
40–59	98.3 (97.1, 99.4)	0.3 (0.04, 0.6)^#^	1.4 (0.2, 2.6)^#^	
60–79	99.0 (98.6, 99.5)	0.5 (0.1, 0.8)^#^	0.5 (0.2, 0.8)^	
Region				
Ontario	93.3 (91.1, 95.5)	1.0 (0.5, 1.4)^	5.7 (3.5, 7.9)^	0.379
Quebec	95.1 (94.1, 96.2)	1.0 (0.7, 1.3)	3.8 (2.7, 4.9)	
Other provinces	93.1 (90.6, 95.6)	1.2 (0.8, 1.7)^	5.6 (3.5, 7.8)^	
Born in Canada				
Yes	93.9 (92.8, 95.0)	1.2 (0.9, 1.6)	4.9 (3.9, 5.9)	0.191
No	93.0 (89.9, 96.1)	0.7 (0.2, 1.1)^	6.3 (3.3, 9.4)^	

^^^ Interpret with caution due to high sampling variability, coefficient of variation (CV) > 16.6% and ≤ 33.3

^#^Interpret with caution due to extreme sampling variability, CV > 33.3%

^a^p-values obtained through chi-squared tests

There was no statistically significant difference in the weighted proportion of specimens with positive, equivocal and negative results by sex, with 93.1% (95% CI: 91.3, 94.9) and 94.3% (95% CI: 92.7, 95.8) positive specimens from males and females, respectively (**Table 3**). There was also no statistically significant difference between the weighted proportions of those born in Canada compared to those born outside Canada, with 93.9% (95% CI: 92.8, 95.0) and 93.0% (95% CI: 89.9, 96.1) of specimens, respectively, above the threshold of protection. Of specimens from Ontario, 93.3% (95% CI: 91.1, 95.5) were above the threshold of protection. For specimens from Quebec and the rest of Canada, 95.1% (95% CI: 94.1, 96.2) and 93.1% (95% CI: 90.6, 95.6) were positive, respectively. There were no statistically significant differences in the proportion of positive, equivocal and negative specimens by geography (**Table 3**).

**Fig 2:**
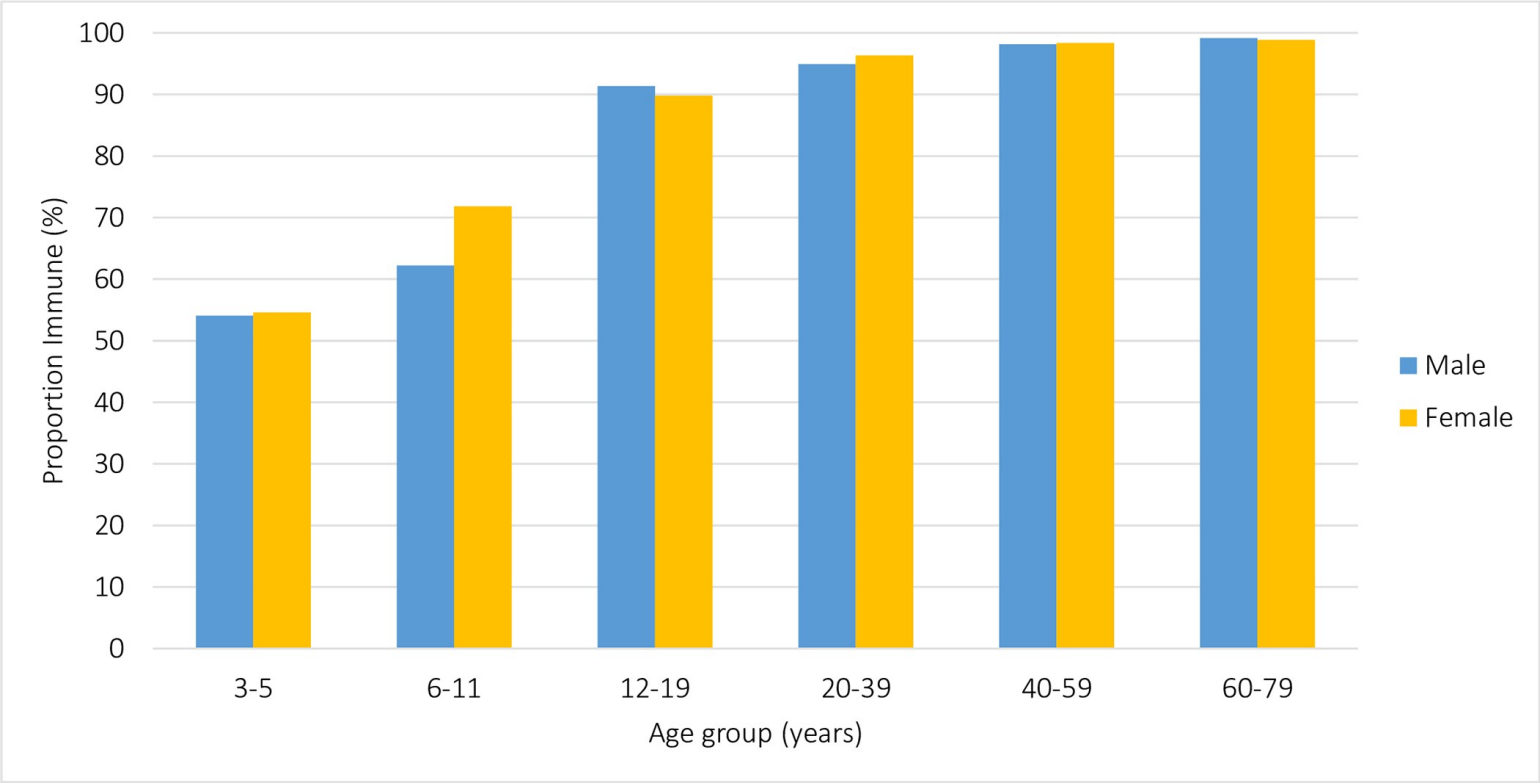
Proportion classified as immune to varicella by sex and age group.

**Fig 3:**
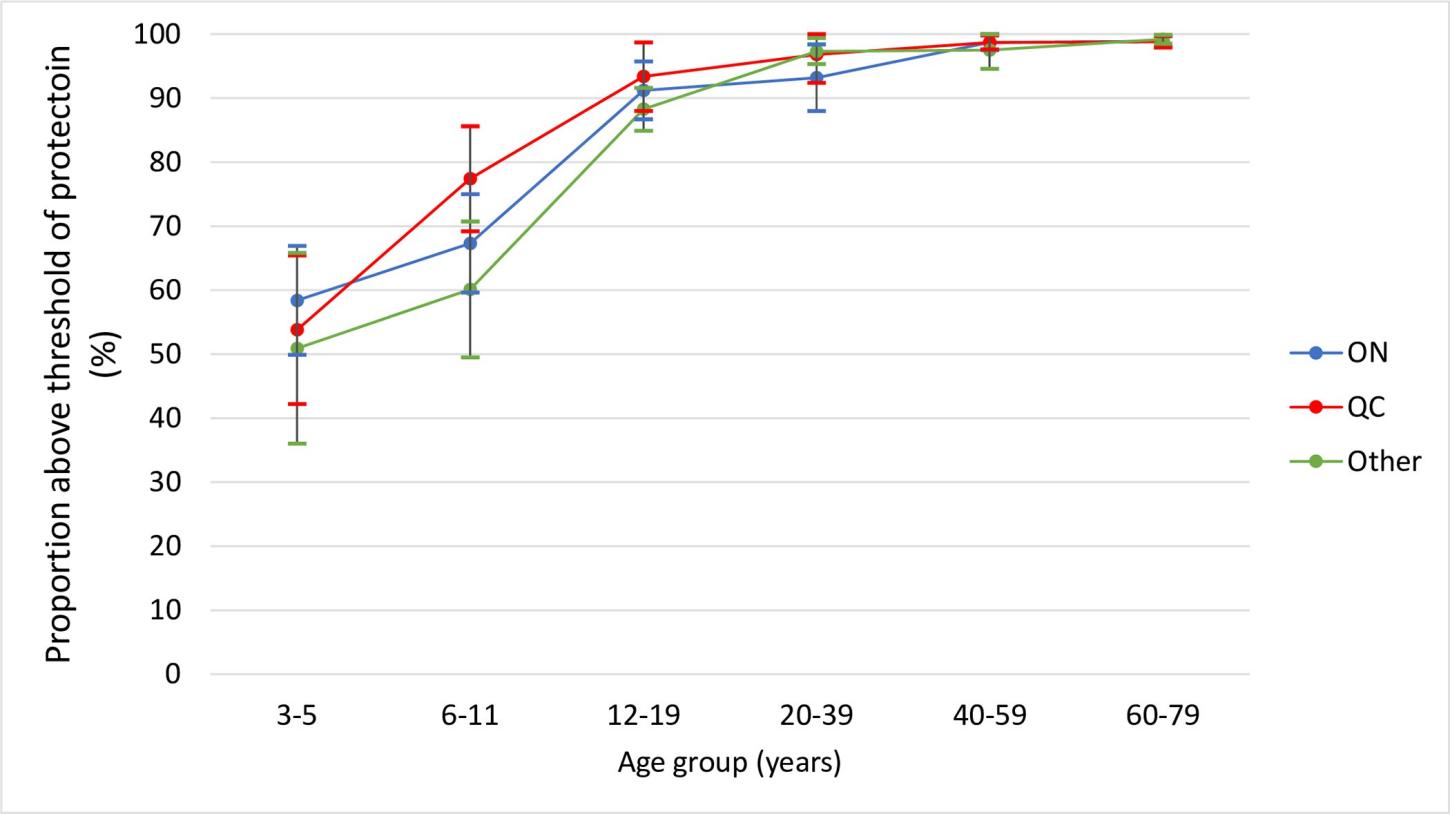
Proportion classified as immune to varicella by region and age group.

**Fig 4:**
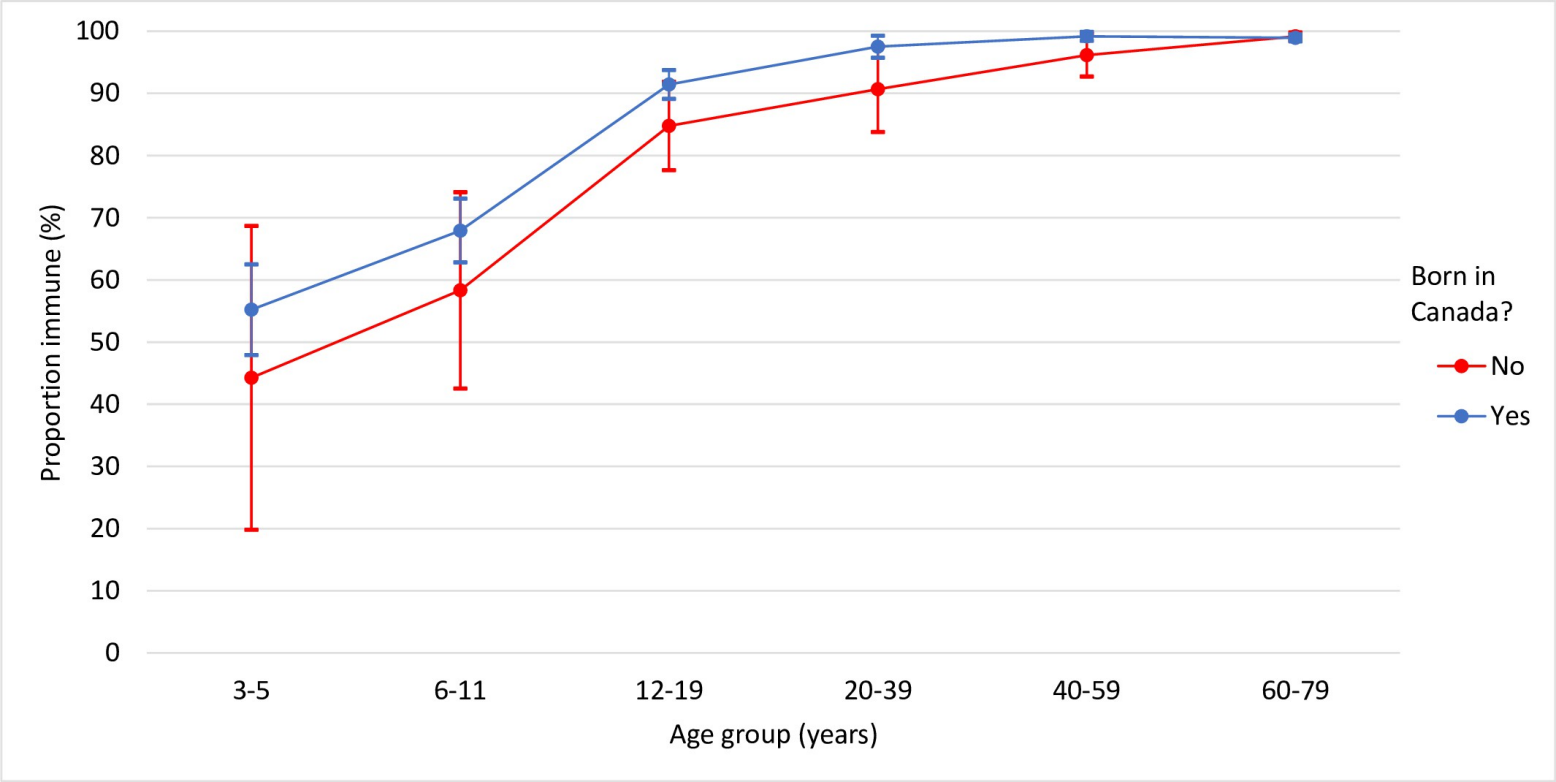
Proportion classified as immune to varicella by country of birth (Canada vs. Other) and age group.

Age-group related positivity trends were consistent across all demographic factors. The proportion of antibodies above the threshold of protection by sex was very similar across age-groups (**Fig 2**). With respect to geographic differences, there was more variation between provinces in the earlier age-groups, though none were statistically significant. Quebec showed a considerable increase in the proportion of children immune from the 3–5 year olds to the 6–11 year olds. From the 20 years of age, there was consistently high immunity across all provinces (**Fig 3**). Finally, individuals born outside Canada had a lower proportion of samples above the threshold of protection than those born in Canada for every age-group except for 60–79 years, though there were no statistically significant differences (**Fig 4**). Adjusting for age-group and sex, individuals not born in Canada had over twice the odds of susceptibility to varicella than those born in Canada (aOR: 2.7; 95% CI: 1.4, 5.0; p<0.01) (**Table 4**).

**Table 4:** Unadjusted and adjusted odds of varicella susceptibility using logistic regression.

	Unadjusted		Adjusted	
	OR (95% CI)	p-value^a^	OR (95% CI)	p-value^a^
Born in Canada				
Yes	REF		REF	
No	1.15 (0.75, 1.78)	0.521	2.66 (1.43, 4.96)	0.004
Sex				
Male	REF		REF	
Female	0.81 (0.58, 1.14)	0.249	0.83 (0.55, 1.25)	0.385
Age-group (years)				
3–5	84.08 (52.15, 135.57)	<0.001	115.58 (64.26, 207.89)	<0.001
6–11	49.75 (28.77, 86.03)	<0.001	65.25 (33.86, 125.74)	<0.001
12–19	10.38 (5.68, 18.95)	<0.001	12.97 (6.52, 25.79)	<0.001
20–39	4.58 (2.03, 10.33)	0.002	4.72 (2.08, 10.73)	0.002
40–59	1.77 (0.82, 3.84)	0.165	1.76 (0.82, 3.80)	0.165
60–79	REF		REF	
Region				
Ontario	REF			
Quebec	0.71 (0.48, 1.06)	0.902		
Other provinces	1.03 (0.61, 1.74)	0.105		

^a^p-values obtained through likelihood ratio tests

Similar to seroprevalence, weighted geometric mean concentrations in CHMS specimens increased with age, with the lowest values in children aged 3–5 years (wGMC: 88 mIU/mL; 95% CI: 60, 117) and 6–11 years (wGMC: 220 mIU/mL; 95% CI: 169, 272). There was a sharp increase in the following age-groups, with the highest values observed in individuals aged 60–79 (wGMC: 1,567 mIU/mL; 95% CI: 1468, 1,666) (**Fig 1**). A test-for-trend showed a highly statistically significant relationship between increasing age-group and wGMC (p<0.0001).

**Fig 5:**
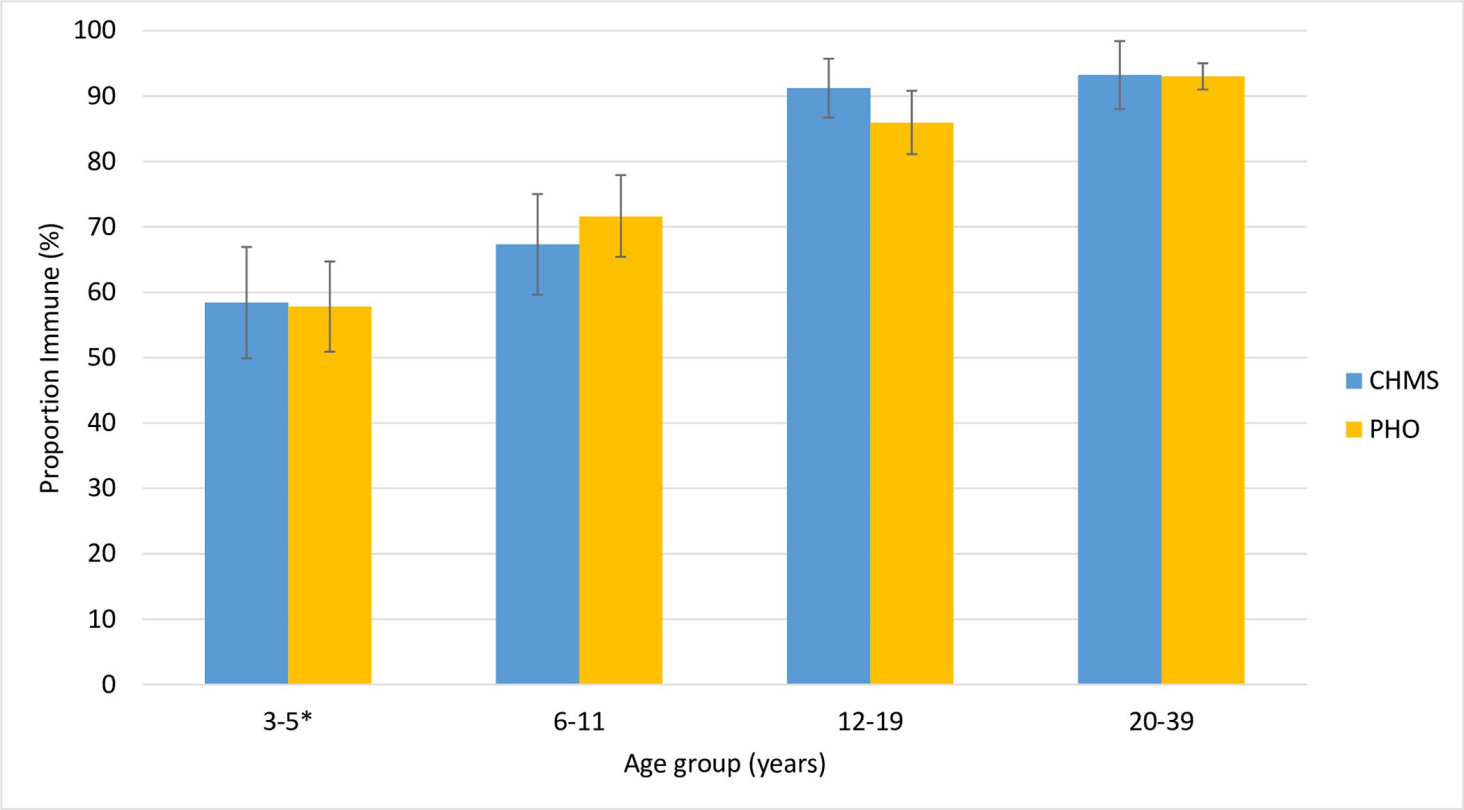
Comparison of immunity to varicella for Ontario as calculated from CHMS samples and Ontario residual specimens. Vertical bars represent 95% Confidence Intervals. *CHMS collected samples from individuals aged 3–5 years, Ontario residual specimens were collected for individuals aged 1–5 years.

Age-related seroprevalence estimates from the 1,199 Ontario residual specimens were very similar to CHMS seroprevalence estimates for Ontario (**Fig 5**), with positivity from both specimen sources rising with age. Seroprevalence was very similar (p = 0.89) between sources for the youngest age-group, at 57.8% (95% CI: 50.9, 64.7) for those aged 1–5 years-old in the residual specimens compared to 58.4% (95% CI: 49.9, 66.9) among 3–5-years-old for CHMS specimens. Residual specimen estimates were slightly higher for 6–11 year olds (71.6%; 95% CI: 65.4, 77.9) than Ontario specimens from CHMS (67.3%; 95% CI: 59.6, 75.0) (p = 0.25). For those aged 12–19, Ontario CHMS estimates (91.2%; 95% CI: 86.7, 95.7) were statistically significantly higher (p = 0.03) than those from residual specimens (85.9%; 95% CI: 81.1, 90.8), although the absolute difference was smaller and in a different direction to 6–11 year olds. Among the last age-group for which both sources had data (20–39 year olds) the proportion of positive specimens was 93.2% (95% CI: 88.0, 98.4) and 93.0% (95% CI 91.0, 95.0) for CHMS and Ontario samples, respectively (p = 0.88).

## Discussion

Our study provides estimates of varicella population immunity in Canadian provinces using data obtained from two different sample sources. Our results demonstrate a lower proportion of individuals with antibodies above the threshold of protection in younger age-groups compared to age-groups that were not vaccine-eligible at the time publicly-funded programs were introduced. The younger age-groups, including almost all children aged 3–5 years, some children aged 6–11 years, and a minority of adolescents aged 12–19 years present in CHMS Cycles 2 and 3, would have been eligible for at least one publicly-funded dose of varicella vaccine (**S2 Table**). Considering this, the lower proportion of specimens with positive results and the high proportion of equivocal results in these age-groups (9.1% in 3–5 year olds and 5.6% in 6–11 year olds, respectively) is not surprising, as they likely represent cohorts not exposed to circulating wild-type VZV.

Our findings are consistent with vaccination coverage estimates for cohorts that were vaccine-eligible soon after program implementation, similar to the cohorts from CHMS Cycles 2 and 3. Although recent Canadian immunization coverage estimates report that 87.5% of two year olds across Canada in 2021 had received at least one dose of varicella-containing vaccine [29], earlier estimates reported lower coverage. For example, Canadian coverage estimates report coverage of only 73.1% for at least one dose for the cohort born in 2011 [30], which was not included in our study. In addition to children who were eligible through the routine immunization program, our study also includes specimens from cohorts that were eligible for one vaccine dose through catch-up campaigns, which had variable timing and uptake depending on the jurisdiction [31, 32]. Lower coverage combined with primary and secondary vaccine failure, which are known characteristics of one-dose VZV vaccination programs [33], may explain our findings.

Our findings indicate that population immunity to varicella in Canada has changed since the pre-vaccine era [34]. Due to lack of vaccination in early childhood, during the pre-vaccine era there was a lower proportion of specimens above the threshold of protection in toddlers and young children compared to those found in our study, with Ratnam *et al*. finding just 33.9% of infants aged four years tested positive for varicella antibody [34] compared to over 54% among those aged 3–5 years in our study. However, there was a subsequent steep rise in immunity in childhood due to natural infection [34]. In our study, we observe a slower increase in the proportion of immune specimens, likely demonstrating high enough vaccine coverage to decrease infection incidence, but not sufficient to achieve robust population immunity levels in early childhood. Accordingly, the median age at infection has risen since the introduction of vaccination programs in parallel with a decrease in incidence [5, 35].

We also demonstrate that seroprevalence estimates derived from residual specimens in one province were comparable to estimates based on specimens collected as part of a nationally-representative sample. The use of residual specimens is believed by some to introduce potential bias, as they may represent individuals who are in poorer health than the general population. This is a particular concern for pediatric specimens. Side-by-side comparisons are rare, but where conducted have shown estimates to be comparable [36]. Conversely, residual specimens for adults sourced from occupational or prenatal screening often represent healthy cohorts. Given the additional time and laboratory resources required to collect specimens and data for the CHMS survey as compared to residual specimens, and the lengthy process enabling us to use them [37, 38], our findings suggest that for some vaccine-preventable diseases, prevalence estimates could be calculated faster and more efficiently using specimens that are already available. However, it is important to understand testing practices for diseases of interest prior to using residual sera. Despite very similar estimates overall, estimated seroprevalence among those aged 12–19 years in CHMS sera was slightly higher than that from the Ontario residual specimens. Since healthy children do not typically have blood taken, it is possible that the residual specimens for individuals in this age-group may not be representative of the general pediatric population, and may be more likely to include those with underlying conditions, which mean they either could not receive vaccination or had a reduced immune response to the vaccinations they received. Additionally, they may have reduced exposure to infection due to different behaviours to those without underlying conditions. Conversely, CHMS may recruit parents who are compliant with regards to immunization, or whose children are less likely to have any underlying condition affecting immunity. However, given the general alignment in findings between the two sources, it appears that any misrepresentation is limited.

There are some limitations to our study. First, the age bands used in the sampling methodology were pre-determined by the CHMS, and therefore did not allow for differentiation between children in the vaccine-eligible cohort and those who are not eligible, or analysis in more granular age-groups. Second, information collected as part of the CHMS did not include individual-level vaccination status for varicella, resulting in interpretation of the data based on vaccine cohort eligibility, and not individual vaccine status. Third, we only assessed a humoral immunity to varicella. Although a humoral correlate of protection has been established for varicella and it is a reliable indication of exposure to vaccination of natural disease, the cell-mediated immune response, which we did not measure, is important for protection from disease [39]. Additionally, individuals not born in Canada but included in the CHMS may not be representative. As immigration from countries with lower seroprevalence and no varicella vaccination increases, the true seroprevalence in these groups may be different to what we calculate here if we do not have a representative sample of people born outside of Canada. Therefore, results relating to those not born in Canada would likely change due to shifts in immigration patterns. Last, the residual sera did not have any associated socio-demographic data which may have allowed for more detailed comparisons with the CHMS results.

Our findings indicate that varicella immunity in Canada is changing, likely associated with the implementation of a routine immunization program. While still generally high for individuals aged 12 and older, the administration of only one vaccine dose in the first years of the publicly- funded program, and limited coverage in catch-up groups, has resulted in low population immunity in younger cohorts. Since varicella remains endemic, these cohorts are at risk of infection and at increased risk of severe disease as they age, especially during pregnancy [40]. Our results underscore the importance of performing periodic serosurveys to monitor further population immunity changes as the proportion of vaccine-eligible birth-cohorts increases, and to continually assess the risk of outbreaks.

## Supporting information

S1 Table BioPlex and gpELISA test results for 11,176 samples from CHMS Cycles 2 and 3 (2009–2013). (DOCX)

S2 Table Summary of varicella vaccine eligibility by age group for age groups represented in CHMS Cycles 2 & 3. (DOCX)
