# Supplementary material for: Population immunity to varicella in Canada: A Canadian Immunization Research Network (CIRN) study
Source: PLoS One. 2024 Aug 19;19(8):e0309154. doi: 10.1371/journal.pone.0309154 (PMC11332944; doi:10.1371/journal.pone.0309154)
Supplement: S1 Table — (DOCX) [file pone.0309154.s001.docx]

|  |  | gpELISA | | | | Total | |
| --- | --- | --- | --- | --- | --- | --- | --- |
|  |  | Positive | Negative | Equivocal | Not tested | n | % |
| BioPlex | Positive |  |  |  | 9296 | 9296 | 83.2 |
|  | Negative | 417 | 1026 | 254 |  | 1697 | 15.2 |
|  | Equivocal | 166 | 1 | 16 |  | 183 | 1.6 |
|  | Total | 583 (5.2%) | 1027 (9.2%) | 270 (2.4%) | 9296 (83.2%) | 11176 | 100 |
