# Supplementary material for: Population immunity to varicella in Canada: A Canadian Immunization Research Network (CIRN) study
Source: PLoS One. 2024 Aug 19;19(8):e0309154. doi: 10.1371/journal.pone.0309154 (PMC11332944; doi:10.1371/journal.pone.0309154)
Supplement: S2 Table — (DOCX) [file pone.0309154.s002.docx]

| **Province** | **Are any individuals from CHMS cycles 2 and 3 eligible for at least one dose of varicella vaccine?** | | | | | |
| --- | --- | --- | --- | --- | --- | --- |
|  | **3-5 years old (birth cohort 2004-2010)** | **6-11 years old (birth cohort 1998-2007)** | **12-19 years old (birth cohort 1990-2001)** | **20-39 years old (birth cohort 1970-1993)** | **40-59 years old (birth cohort 1950-1973)** | **60+ years old** |
| **Prince Edward Island** | YES | YES | MINORITY | NO | NO | NO |
| **Alberta** | YES | YES | MINORITY | NO | NO | NO |
| **Nova Scotia** | YES | YES | MINORITY | NO | NO | NO |
| **Ontario** | YES | YES | NO | NO | NO | NO |
| **New Brunswick** | YES | YES | NO | NO | NO | NO |
| **Manitoba** | YES | YES | NO | NO | NO | NO |
| **Newfoundland and Labrador** | YES | YES | NO | NO | NO | NO |
| **Saskatchewan** | YES | YES | NO | NO | NO | NO |
| **British Columbia** | YES | YES | NO | NO | NO | NO |
| **Quebec** | YES | MINORITY | NO | NO | NO | NO |
| **Northwest Territories** | YES | YES | NO | NO | NO | NO |
| **Yukon** | YES | MINORITY | NO | NO | NO | NO |
| **Nunavut** | YES | YES | NO | NO | NO | NO |
